# Supplementary figures and images for: ER-dependent membrane repair of mycobacteria-induced vacuole damage
Source: mBio. 2023 Sep 7;14(5):e00943-23. doi: 10.1128/mbio.00943-23 (PMC10653851; doi:10.1128/mbio.00943-23)

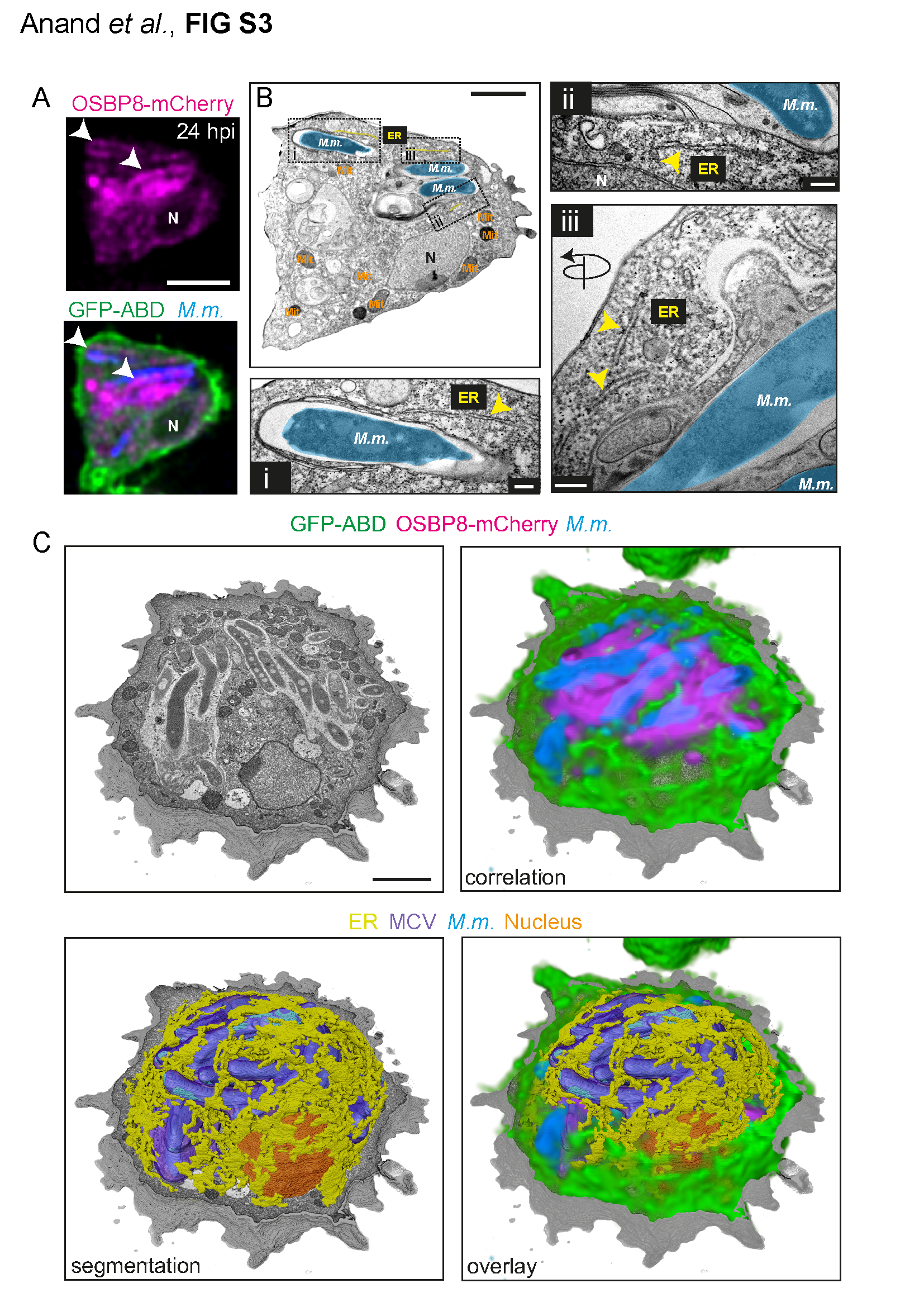

Supplement: Figure S3 — Correlative ultrastructural analysis to monitor OSBP8-mCherry+ ER-tubules in close proximity to the MCV. [file mbio.00943-23-s0003.tif]
