# Supplementary material for: ER-dependent membrane repair of mycobacteria-induced vacuole damage
Source: mBio. 2023 Sep 7;14(5):e00943-23. doi: 10.1128/mbio.00943-23 (PMC10653851; doi:10.1128/mbio.00943-23)
Supplement: Legends — Supplemental figure legends. [file mbio.00943-23-s0007.docx]

**“ER-dependent membrane repair of mycobacteria-induced vacuole damage”**

Aby Anand, Anna-Carina Mazur, Patricia Rosell-Arevalo, Rico Franzkoch, Leonhard Breitsprecher, Stevanus A. Listian, Sylvana V. Hüttel, Danica Müller, Deise G. Schäfer, Simone Vormittag, Hubert Hilbi, Markus Maniak, Maximiliano G. Gutierrez and Caroline Barisch^*^

**Supplemental Material**

**FIG S1** Mycobacterial infection induces ER-MCV contacts. (A) ER in apposition with the MCV. Representative images of cells showing Calnexin-mCherry^+^ ER-tubules close to the MCV (arrow heads). Cells were infected with eBFP-expressing *M. marinum*. At 24 hpi cells were fixed and stained for p80 to label the membrane of the MCV. Scale bars, 5 µm; Zoom, 2 µm. (B) Domain organization of OSBPs in *D. discoideum* (*Dd*) compared to long (ORP1L, Osh1) and short ORP family members from *Homo sapiens* (*Hs*) and *Saccharomyces cerevisiae* (*Sc*) (ORP1S, Osh4). PH: pleckstrin homology; FFAT: two phenylalanines (FF) in an acidic tract. (C) Conserved fingerprint sequence of *D. discoideum* OSBPs.

**FIG S2** Localization of OSBP8 in non-infected cells and of OSBP7 in infected cells. (A-B) Subcellular localization of OSBP8-GFP. Cells overexpressing OSBP8-GFP/Calnexin-mCherry or OSBP8-GFP/ZntC-mCherry were imaged live by SD microscopy. Arrow points to the juxtanuclear region or the Golgi-apparatus. Scale bars, 5 µm. Images were deconvolved. (C-D) Subcellular localization of GFP-OSBP8. (E-F) OSBP8-GFP localization on BCPs. Cells overexpressing OSBP8-GFP or GFP-OSBP8 were incubated with fluorobeads for 2 hrs, fixed and then stained with αVatA (vATPase subunit A, lysosomes) and αVacA (VacuolinA, post-lysosomes) antibodies. Arrows point to VatA^+^ or VacA^+^ BCPs. Asterisks indicate fluorobeads, N: nucleus. Scale bars, 5 µm. (G) Mobilization of OSBP7-GFP upon infection. Cells overexpressing OSBP7-GFP were infected with mCherry-expressing *M. marinum*. At the indicated time points, cells were imaged live by SD microscopy. Arrow heads indicate OSBP7-GFP^-^ mycobacteria. Scale bars, 5 µm; Zoom, 2 µm.

**FIG S3.** Correlative ultrastructural analysis to monitor OSBP8-mCherry^+^ ER-tubules in close proximity to the MCV. (A-B) CLEM images showing OSBP8-mCherry at ER-MCV contacts (white arrow heads). Cells expressing OSBP8-mCherry/GFP-ABD were infected with eBFP-expressing *M. marinum*. At 24 hpi, cells were imaged after quick fixation by SD microscopy (A), high pressure frozen and prepared for EM (B). Positions of the closeups are indicated. (i - iii) Closeups showing OSBP8-mCherry^+^ ER-tubules close to the MCV. Yellow arrowheads point to ER-tubules in the vicinity of *M. marinum*. Mitochondria (Mit) were indicated in orange text, *M. marinum* (*M.m*.) and ER-tubules are pseudo-coloured in cyan and in yellow, respectively. N: nucleus. Scale bars, 5 µm (A); 2 µm (B) and 200 nm (i - iii). SD images were deconvolved. (C) SBF-SEM-derived images illustrate the correlation of the SD images and the volumetric segmentation of the EM data shown in Fig. 2F-G. The MCV is segmented in violet, *M. marinum* (*M.m*.) in cyan, ER-tubules in yellow and the nucleus in orange. Scale bars, 2 µm.

**FIG S4** Subcellular localization of endogenous OSBP8::GFP during infection with *M. marinum* wt and ΔRD1. (A) Expression levels of OSBP8-GFP (overexpressed) compared to OSBP8::GFP (endogenous). (B) Quantification of A. Cells expressing OSBP8-GFP as well as OSBP8::GFP were harvested and then prepared for western blotting. The intensity of the bands was measured using ImageJ. AUC: area under the curve. (C-D) OSBP8::GFP mobilization during infection. Cells expressing OSBP8::GFP were infected with eBFP-expressing *M. marinum* wt or ΔRD1. At the indicated time points samples were taken for SD microscopy. Arrows point to OSBP8::GFP^+^ intracellular mycobacteria and arrow heads indicate OSBP8::GFP^-^ mycobacteria. Scale bars, 5 µm; Zoom, 2,5 µm. Images were deconvolved. *M.m.:* *M.* *marinum.*

**FIG S5** Localization of GFP-Vps32, P4C-GFP, OSBP8-GFP and OSBP7-GFP in non-infected cells. (A) Dynamics of GFP-Vps32, P4C-GFP, OSBP8-GFP and OSBP7-GFP on damaged lysosomes. (B-C) Recruitment of OSBP8-GFP to damaged lysosomes in cells highly expressing P4C-mCherry. Cells expressing GFP-Vps32, P4C-GFP, OSBP8-GFP, OSBP7-GFP or P4C-mCherry/OSBP8-GFP were incubated overnight with 10 kDa fluorescent dextran to label all endosomal compartments and then subjected to LLOMe. Arrow heads point to GFP-Vps32^+^, P4C-GFP^+,^ P4C-mCherry^+^, OSBP8-GFP^+^ or OSBP7-GFP^-^ lysosomes. Scale bars, 5 µm; Zoom, 1 µm. (D) OSBP8-GFP recruitment to MCVs in cells expressing P4C-mCherry at low levels. Cells overexpressing OSBP8-GFP/P4C-mCherry were infected with eBFP-expressing *M. marinum* wt. Images were taken at 3 and 46 hpi. Arrow heads point to OSBP8-GFP^+^ *M. marinum*. Scale bars, 5 µm. *M.m.:* *M.* *marinum.*

**FIG S6** Effect of OSBP8 depletion on PI4P distribution and cell viability during lysosomal damage and on vacuolar escape of mycobacteria during infection. (A) PI4P distribution in non-infected wt and cells lacking OSBP8. (B) Quantification of A. Wt and *osbH* KO cells were imaged live. Shown are maximum z-projections of 15 z-stacks 300 nm apart. Scale bar, 5 µm. To label the PM for the quantification in B, cells were pre-stained with FM4-64. For each condition 108 cells per cell line were quantified using ImageJ. Plots show the mean and standard deviation of three independent experiments. Statistical significance was calculated with an unpaired t-test (***, p < 0.001). (C) P4C-GFP localization on damaged lysosomes in cells lacking OSBP8. Wt and *osbH* KO cells expressing P4C-GFP were incubated overnight with 10 kDa fluorescent dextran to label all endosomal compartments and then treated with LLOMe. Images were recorded every 5 min by SD microscopy. GFP-signal of the *osbH* KO cells was enhanced for better visualization. Scale bars, 5 µm. (D) Cell viability in wt and cells lacking OSBP8 upon LLOMe treatment. Wt, *osbH* or *tsg101* KOs were labelled with propidium iodide (PI) and incubated with LLOMe for 60 min. 10,000 cells were analysed per condition. Graphs are representative for two independent experiments. (E) Quantification of D. Plots were gated as indicated in (D), to reveal the number of dead cells. Plots in (E) show the mean and standard deviation of three independent experiments. Statistical differences were calculated with an unpaired t-test (*, p < 0.05; **, p < 0.01). (F) Vacuolar escape of mycobacteria in wt and cells lacking OSBP8. (G) Percentage of ubiquitin^+^ bacteria in wt and *osbH* KOs at 8 and 21 hpi. Wt and *osbH* KO cells were infected with mCherry-expressing *M. marinum*, fixed and stained against ubiquitin (FK2) (green) and p80 (magenta). Representative maximum projections of 5 z-stacks of 500 nm at 21 hpi is shown in E. White arrowheads label ubiquitinated bacteria. Scale bars, 10 μm; Plots in (G) show the mean and standard deviation of three independent experiments (FK2 8, 21 hpi N=3, 138≤n≤462). Statistical differences were calculated with an unpaired t-test. ns: not significant. *M.m.:* *M.* *marinum.* (H) OSBP8 contains an unstructured N-terminus as well as an ALPS-like motif. The OSBP8 structure was derived from AlphaFold (https://alphafold.ebi.ac.uk/entry/Q54QP6) and analysed using HeliQuest (https://heliquest.ipmc. cnrs.fr/).

**Movie S1** Monitoring OSBP8-GFP recruitment by LLSM. For more information see Fig. 2A-B.

**Movie S2** 3D rendering of LLSM data. For more information see Fig. 2C.

**Movie S3** Correlation and 3D reconstruction of SBF-SEM data. For more information see Fig. 2E-G, Fig. S3C.
