# Supplementary material for: ER-dependent membrane repair of mycobacteria-induced vacuole damage
Source: mBio. 2023 Sep 7;14(5):e00943-23. doi: 10.1128/mbio.00943-23 (PMC10653851; doi:10.1128/mbio.00943-23)
Supplement: TABLE S1 — Material used in this publication. [file mbio.00943-23-s0011.docx]

**Table S1** Material used in this publication.

| ***D. discoideum* Strains** | **Relevant characteristics** | **Source/Reference** |
| --- | --- | --- |
| Ax2 | wt, parental strain of *osbH* KO, *osbH* KI |  |
| Ax2 *osbH* KO | Bsr^r^ | This study |
| Ax2(Ka) | wt, parental strain of *tsg101* KO |  |
| Ax2(Ka) *tsg101* KO | Bsr^r^ | ([21](#_ENREF_21)) |
| ***D. discoideum* Plasmids** |  | **Source/Reference** |
| OSBP8-GFP | pDM323-*osbH*, G418^r^, Amp^r^ | This study |
| GFP-OSBP8 | pDM317-*osbH*, G418^r^, Amp^r^ | This study |
| Calnexin-mCherry | pDM1044-*Calnx*, Hyg^r^, Amp^r^ | ([23](#_ENREF_23)) |
| ZntD-mCherry | pDM1044-*zntD*, Hyg^r^, Amp^r^ | ([21](#_ENREF_21)) |
| AmtA-mCherry | pDM1044-*amtA*, Hyg^r^, Amp^r^ | ([18](#_ENREF_18)) |
| OSBP8-mCherry | pDM1210-*osbH*, Hyg^r^, Amp^r^ | This study |
| OSBP7-GFP | pDM323-*osbG*, G418^r^, Amp^r^ | This study |
| P4C-mCherry | pDM1044-*p4c*, Hyg^r^, Amp^r^ | ([20](#_ENREF_20)),([23](#_ENREF_23)) |
| P4C-GFP | pDM323-*p4c*, G418^r^, Amp^r^ | ([46](#_ENREF_46)) |
| GFP-Vps32 | pDM317-*vps32*, G418^r^, Amp^r^ | ([6](#_ENREF_6)) |
| OSBP8::GFP | pPI183-osbH, Hyg^r^, Amp^r^ | This study |
| GFP-ABD | pDXA-GFP-ABD120, G418^r^, Amp^r^ | ([47](#_ENREF_47)) |
| **Mammalian cells** | **Relevant characteristics** | **Source/Reference** |
| Human induced pluripotent stem cell-derived macrophages (iPSDMs) | iPSDMs were generated from human induced pluripotent stem cell line KOLF2 | Public Health England Culture Collections (catalogue number 77650100) |

| *M. marinum* material |  |  |
| --- | --- | --- |
| *M. marinum* M | wt, parental strain | L. Ramakrishnan (University of Cambridge) |
| *M. marinum* ΔRD1 | RD1 locus deletion mutant | L. Ramakrishnan (University of Cambridge) ([48](#_ENREF_48)) |
| *M. marinum* ΔCE | *esxA* and *esxB* deletion mutant | T. Soldati (University of Geneva) ([28](#_ENREF_28)) |
| *M. tuberculosis* material |  |  |
| *M. tuberculosis* | H37Rv | Douglas Young (The Francis Crick Institute, London, UK), ([13](#_ENREF_13)) |
| *M. tuberculosis* ΔRD1 | H37Rv ΔRD1 | Suzie Hingley-Wilson (University of Surrey, Guilford, UK), ([13](#_ENREF_13)) |
| Mycobacteria Plasmids |  |  |
| pTEC18 | eBFP2 under control of the MSP promoter, Hyg^r^, Amp^r^ | Addgene #30177([49](#_ENREF_49)) |
| pCherry10 | mCherry under control of the G13 promoter, Hyg^r^, Amp^r^ | Addgene #24664 ([50](#_ENREF_50)) |
| pTEC19 | E2-Crimson under the control of the MSP promotor, Hyg^r^, Amp^r^ | Addgene #30178 ([49](#_ENREF_49)) |

6. López-Jiménez AT, Cardenal-Muñoz E, Leuba F, Gerstenmaier L, Barisch C, Hagedorn M, King JS, Soldati T. 2018. The ESCRT and autophagy machineries cooperate to repair ESX-1-dependent damage at the Mycobacterium-containing vacuole but have opposite impact on containing the infection. PLoS Pathog 14:e1007501. <https://doi.org/10.1371/journal.ppat.1007501>

13. Bernard EM, Fearns A, Bussi C, Santucci P, Peddie CJ, Lai RJ, Collinson LM, Gutierrez MG. 2020. M. tuberculosis infection of human iPSC-derived macrophages reveals complex membrane dynamics during xenophagy evasion. J Cell Sci 134. <https://doi.org/10.1242/jcs.252973>

18. Barisch C, Paschke P, Hagedorn M, Maniak M, Soldati T. 2015. Lipid droplet dynamics at early stages of Mycobacterium marinum infection in Dictyostelium. Cell Microbiol 17:1332-49. <https://doi.org/10.1111/cmi.12437>

20. Vormittag S, Hüsler D, Haneburger I, Kroniger T, Anand A, Prantl M, Barisch C, Maaß S, Becher D, Letourneur F, Hilbi H. 2023. Legionella- and host-driven lipid flux at LCV-ER membrane contact sites promotes vacuole remodeling. EMBO Rep doi:10.15252/embr.202256007:e56007. <https://doi.org/10.15252/embr.202256007>

21. Barisch C, Kalinina V, Lefrançois LH, Appiah J, López-Jiménez AT, Soldati T. 2018. Localization of all four ZnT zinc transporters in Dictyostelium and impact of ZntA and ZntB knockout on bacteria killing. J Cell Sci 131. <https://doi.org/10.1242/jcs.222000>

23. Steiner B, Swart AL, Welin A, Weber S, Personnic N, Kaech A, Freyre C, Ziegler U, Klemm RW, Hilbi H. 2017. ER remodeling by the large GTPase atlastin promotes vacuolar growth of Legionella pneumophila. EMBO Rep 18:1817-1836. <https://doi.org/10.15252/embr.201743903>

28. Cardenal-Munoz E, Arafah S, Lopez-Jimenez AT, Kicka S, Falaise A, Bach F, Schaad O, King JS, Hagedorn M, Soldati T. 2017. Mycobacterium marinum antagonistically induces an autophagic response while repressing the autophagic flux in a TORC1- and ESX-1-dependent manner. PLoS Pathog 13:e1006344. <https://doi.org/10.1371/journal.ppat.1006344>

46. Welin A, Weber S, Hilbi H. 2018. Quantitative Imaging Flow Cytometry of Legionella-Infected Dictyostelium Amoebae Reveals the Impact of Retrograde Trafficking on Pathogen Vacuole Composition. Appl Environ Microbiol 84. <https://doi.org/10.1128/AEM.00158-18>

47. Pang KM, Lee E, Knecht DA. 1998. Use of a fusion protein between GFP and an actin-binding domain to visualize transient filamentous-actin structures. Curr Biol 8:405-8. <https://doi.org/10.1016/S0960-9822(98)70159-9>

48. Volkman HE, Clay H, Beery D, Chang JC, Sherman DR, Ramakrishnan L. 2004. Tuberculous granuloma formation is enhanced by a mycobacterium virulence determinant. PLoS Biol 2:e367. <https://doi.org/10.1371/journal.pbio.0020367>

49. Takaki K, Davis JM, Winglee K, Ramakrishnan L. 2013. Evaluation of the pathogenesis and treatment of Mycobacterium marinum infection in zebrafish. Nat Protoc 8:1114-24. <https://doi.org/10.1038/nprot.2013.068>

50. Carroll P, Schreuder LJ, Muwanguzi-Karugaba J, Wiles S, Robertson BD, Ripoll J, Ward TH, Bancroft GJ, Schaible UE, Parish T. 2010. Sensitive detection of gene expression in mycobacteria under replicating and non-replicating conditions using optimized far-red reporters. PLoS One 5:e9823. <https://doi.org/10.1371/journal.pone.0009823>
